# Multi-omics analysis reveals STING activation mediates NLRP3-related pyroptosis and exacerbates myocardial ischemia-reperfusion injury

**DOI:** 10.1371/journal.pone.0341839

**Published:** 2026-02-06

**Authors:** Xuehu Zhang, Aoqin Gu, Lirong Zhou, Yuru Ma, Peng Wu, Baozhen Zhu, Ru Yan, Guangzhi Cong, Xueping Ma, Shaobin Jia

**Affiliations:** 1 Heart Center & Department of Cardiovascular Diseases, General Hospital of Ningxia Medical University, Yinchuan, Ningxia, China; 2 Ningxia Medical University, Yinchuan, Ningxia, China; Qinghai University, CHINA

## Abstract

**Background:**

Myocardial ischemia-reperfusion injury (I/R) significantly exacerbates cardiomyocyte damage post-recanalization therapy in acute myocardial infarction. Pyroptosis and the NLRP3 inflammasome are crucial in I/R, yet the precise mechanism remains unclear.

**Materials and Methods:**

Transcriptomics, proteomics, and single-cell transcriptomics were employed to examine cellular subtype changes and pyroptosis-associated gene regulation in I/R. Differential pyroptosis-related genes were identified from transcriptomics data and validated with proteomics. Single-cell RNA sequencing assessed pyroptosis levels and intercellular communication. Mouse myocardial I/R and human cardiomyocyte hypoxia/reoxygenation (H/R) models were used to explore STING overexpression/silencing effects on NLRP3 inflammasome activation, oxidative stress, and cellular injury.

**Results:**

Pyroptosis-related genes were significantly dysregulated, implicating multiple inflammatory pathways. Single-cell analyses revealed increased granulocytes, macrophage infiltration, cardiomyocyte injury, and enhanced pyroptosis scores post-I/R. Cardiomyocytes, endothelial cells, and fibroblasts exhibited increased pyroptosis and inflammatory cell-cell interactions. Animal studies indicated significant declines in cardiac function and increased oxidative stress and inflammation post-I/R. STING activation (SR717) worsened cardiac function, enhanced ROS production, and elevated myocardial injury markers; STING inhibition (H151) markedly mitigated these effects. Correspondingly, the cGAS-STING pathway and NLRP3 inflammasome factors were significantly upregulated post-I/R, exacerbated by STING agonists and alleviated by STING inhibitors. Cellular studies confirmed that STING overexpression intensified oxidative stress and pyroptosis, effects reversed by STING knockdown and blocked by NLRP3 inhibitor MCC950.

**Conclusion:**

STING activation contributes to oxidative stress and NLRP3 inflammasome–associated pyroptosis during I/R. Targeting the STING-NLRP3 axis may represent a potential strategy to reduce myocardial injury after ischemia-reperfusion.

## Introduction

Acute myocardial infarction (AMI) is one of the leading causes of death and disability worldwide [1]. Prompt restoration of perfusion to the ischemic region is the most effective treatment for AMI, but the subsequent myocardial ischemia-reperfusion injury (I/R) can cause further damage to cardiomyocytes [2,3]. Multiple regulated cell death programs contribute to I/R injury, including apoptosis, autophagy-dependent cell death, pyroptosis, and ferroptosis [4,5]. Because these pathways interact and amplify each other, effective interventions for I/R remain limited; therefore, identifying key upstream regulators and their downstream effector pathways is important for developing preventive and therapeutic strategies.

NLRP3 inflammasome is a multiprotein complex assembled predominantly by the NOD-like receptor family pyrin domain-containing 3 (NLRP3), which recognizes a variety of pathogen-associated or damage-associated molecular patterns that activate downstream inflammatory effects [6]. During I/R, a large number of damage-associated molecular patterns (DAMP) trigger activation of NLRP3 inflammasome, leading to the maturation and release of the pro-inflammatory cytokines interleukin-1β (IL-1β) and interleukin-18 (IL-18). These factors not only exacerbate the local inflammatory response, but also induce myocardial cells to undergo pyroptosis, thereby damaging myocardial tissues [7].Meng et al. [8] showed that NLRP3 inflammasome–associated factors were significantly upregulated in an I/R mouse model. Inhibition of NLRP3 inflammasome activity attenuated inflammation, reduced infarct size, and improved cardiac function. The NLRP3 inflammasome exacerbates myocardial injury during I/R by triggering gasdermin D (GSDMD)–mediated pyroptosis [9]. In contrast, the NLRP3 inflammasome inhibitor MCC950 attenuates myocardial fibrosis and improves cardiac function in I/R by inhibiting the early inflammatory response [10]. Therefore, clarifying upstream regulators and downstream effectors of the NLRP3 inflammasome is important for developing targeted interventions in I/R.

Stimulator of interferon genes (STING) is an endoplasmic reticulum–resident adaptor protein that plays a central role in innate immune signaling [11]. In the canonical cGAS–STING pathway, cyclic GMP–AMP synthase (cGAS) senses cytosolic DNA and generates cyclic GMP–AMP (cGAMP). cGAMP binds and activates STING, which dimerizes and translocates to recruit TANK-binding kinase 1 (TBK1) and phosphorylate IRF3, thereby inducing type I interferons and other inflammatory mediators [12–14]. The role of the STING pathway in cardiovascular disease has received increasing attention in recent years. Myocardial infarction leads to ischemic necrosis of cardiomyocytes, resulting in rupture of the cell membrane and release of intracellular and mitochondrial DNA into the cytoplasm [15]. This cytoplasmic DNA is recognized by intracellular cGAS, which activates STING. activated STING promotes the expression of type I interferons (e.g., IFN-β) and proinflammatory cytokines (e.g., TNF-α, IL-6, and IL-1β), which further activate local and systemic immune responses [16]. The cGAS–STING pathway can recruit inflammatory cells (e.g., neutrophils, macrophages, and monocytes) to the infarcted area, increasing local inflammatory mediators. Sustained activation may also promote myocardial fibrosis through prolonged inflammation [17]. Activation of the cGAS–STING axis has been reported to drive neuroinflammation and induce inflammasome activation and microglial pyroptosis in a cerebral venous sinus thrombosis (CVST) mouse model [18]. The mtDNA–cGAS–STING axis contributes to sepsis-induced acute kidney injury through activation of the NLRP3 inflammasome [19]. In addition, myeloid SHP2 has been reported to attenuate myocardial I/R injury by regulating BRD4/SYK/STING/NOX4/NLRP3 signaling [20]. Taken together, these studies suggest that STING may contribute to myocardial I/R injury, at least in part, through NLRP3 inflammasome activation; however, the precise regulatory mechanisms in cardiomyocytes and in the I/R microenvironment remain incompletely defined.

Bioinformatics analyses can facilitate efficient identification of key genes and pathways. In this study, we focused on the STING/NLRP3 axis to investigate its role in myocardial I/R injury. We integrated bulk transcriptome analyses with single-cell data to characterize dysregulated pyroptosis-related genes and their cellular distribution during I/R. We then modulated STING activity in a mouse I/R model and a cellular H/R model to evaluate NLRP3 inflammasome activation, oxidative stress, and cell survival. These results provide experimental evidence supporting myocardial protection strategies targeting STING and/or the NLRP3 inflammasome.

## Materials and methods

### Screening and characterization of pyroptosis -related differential genes

Myocardial ischemia/reperfusion-related transcriptome expression profiling datasets were obtained from the Gene Expression Omnibus (GEO) database. Data containing ischemia/reperfusion model groups and controls were selected, and the gene expression matrices were background-corrected and normalized based on probe information. The following three datasets were used; GSE83472 (control group n = 4, disease group n = 4); GSE61592 (control group n = 3, disease group n = 3); GSE160516 (control group n = 4 individuals, disease group n = 12). The sva R [21] package was used for batch correction. limma [22]package was used for differential expression analysis. Then, a total of 622 genes (Relevance score ≥ 1.0) were screened for the list of genes related to cellular pyroptosis based on the reported gene functions in the GeneCards database (https://www.genecards.org/) included in the literature (**S1 Table**). The entire study utilized a merged dataset comprising the following three datasets, which underwent batch correction prior to subsequent analysis. Human gene symbols associated with pyroptosis obtained from GeneCards were converted to mouse orthologues, then intersected with the mouse GEO expression matrix. Differentially expressed genes were selected based on the criteria |log2FC| > 0.263 and adj. p < 0.05. The intersection of the differential genes with the list of pyroptosis-related genes was taken to obtain the set of pyroptosis-related differential genes in I/R.

### Analysis of single-cell RNA sequencing data

Single-cell RNA sequencing data were obtained from publicly available myocardial tissue samples from the mouse cardiac I/R model (GSE146285). Raw Data are from Molenaar et al. and reanalyzed by us [23]. A total of 8,970 cells were obtained, with an average of 2,768 genes detected per cell. The single-cell data were analyzed by quality control, normalization and dimensionality reduction using the Seurat [24] software package. After quality control (excluding cells with fewer than 200 or more than 7,500 genes; cells with fewer than 500 or more than 50,000 UMIs; cells with mitochondrial gene content exceeding 25%; and cells with hemoglobin gene content exceeding 10%), a total of 2,245 cells were retained. And then downscaled by PCA and classified by clustering based on significant principal components, and cells were visualized in two-dimensional space using a t-SNE-based approach. The major cell groups such as cardiomyocytes, fibroblasts, endothelial cells, macrophages, granulocytes, etc. were obtained by annotation based on cell type-specific marker gene expression in SingleR [25]. The celldex [25] reference dataset MouseRNAseqData (1.18) was used as the main reference for annotation. Next, the obtained set of pyroptosis signature genes was used to calculate the pyroptosis score for each single cell: the expression of pyroptosis-related genes at the single-cell level was synthesized into a single score using the AddModuleScore function. The distribution of the pyroptosis scores of different cell types was compared to identify cell populations with higher pyroptosis activity in the ischemic injury environment. Cellular communication analysis of the single-cell transcriptome was performed using the CellChat [26] tool. Significant signaling pathways and ligand-receptor pairs between different cell types were predicted based on ligand-receptor interaction databases, and a network map of intercellular communication within cardiac tissues was constructed. The role of highly pyroptosis-active cell populations in the network and the strength of their interactions with cardiomyocytes were assessed.

### Mouse myocardial I/R model construction

C57BL/6J mice were provided by the Experimental Animal Center of Ningxia Medical University and fed with mouse-specific food provided by the center. Healthy 8-week-old SPF-grade male mice were selected, with a body weight range of 20−25 g. The mice were fasted for 12 hours before the experiment, but provided with sufficient water. Ethics committee of Ningxia Medical University approved the experiment (KYLL-2024–0873). All experiments were performed in accordance with relevant guidelines and regulations. Forty C57BL/6J mice were randomly divided into the following five groups: the Sham group, the I/R group, the I/R + SR717 (STING agonist) group, the I/R + H151 (STING inhibitor) group, and the I/R+DMSO group, with 8 mice in each group. Numbering was performed with ear tags. The I/R model [27] was established, and the Sham group was operated as the I/R group except that the coronary blood flow was not blocked. The I/R + SR717 group and the I/R + H151 group were given two intraperitoneal injections of SR717 (10 mg/kg) [28] or H151 (3 mg/kg) 48 and 24 hours before ischemia, respectively [29], and the I/R+DMSO group was injected with equal doses of DMSO (10 mL/kg) at the same time points. Immediately after ischemia, the I/R + SR717 group, the I/R + H151 group, and the I/R+DMSO group were again injected once with the above drugs in each group. Myocardial ischemia/reperfusion procedures were referred to previously reported methods [4,30]. Body weight of the mice were monitored weekly throughout the study. No mice were excluded from the study, and none of the animals showed any serious clinical signs throughout the experiment. At the end of the experiment, the mice were immediately euthanized by cervical dislocation.

### Proteomic detection and analysis of mouse I/R left ventricular myocardial tissue

Proteins were extracted from mouse cardiac tissue homogenates using ice-cold lysis buffer, sonicated, centrifuged, and quantified by BCA assay. Samples underwent reduction (DTT, 56°C) and alkylation (iodoacetamide, dark incubation), followed by urea dilution (<2M). Proteins were digested sequentially with trypsin overnight and again at a 1:100 ratio for 4 hours. Digested peptides were separated via high-performance liquid chromatography (Agilent 300 Extend C18 column) using a gradient elution (6–80% mobile phase B over 60 min). Peptides were analyzed using LC-MS/MS with nanospray ionization and data-dependent acquisition (DDA), selecting the top 20 precursor ions for fragmentation. Raw mass spectrometry data were processed using MaxQuant software, normalized, log2-transformed, and analyzed statistically. Differentially expressed proteins were identified (FC ≥ 1.2, P < 0.05) and functionally annotated using GO and KEGG pathway enrichment analyses.

### Histopathology and ultrastructural testing

1) Hematoxylin-eosin (HE) staining:

Cardiac tissues were taken quickly after mouse execution, fixed with 4% paraformaldehyde overnight, dehydrated with gradient ethanol and paraffin embedded after xylene transparency. 4 µm thick paraffin sections were made, deparaffinized and gradient hydrated, stained with hematoxylin and eosin, differentiated with acidic alcohol and rinsed with sufficient running water, dehydrated with gradient ethanol, sealed with xylene clear, and myocardial histopathological alterations were observed under the microscope.

2) Immunofluorescence staining of left ventricular myocardium:

Paraffin sections were dewaxed and hydrated, permeabilized with 0.5% Triton, washed with PBS and sealed with goat serum. STING and NLRP3 primary antibodies were added and incubated at 4°C overnight (IL-1β and IL-18 primary antibodies were used for cellular experiments), washed with PBS on the next day, and the fluorescent secondary antibody was incubated at room temperature and protected from light for 1 hour, then the sections were blocked with DAPI blocking agent, and the sections were viewed by fluorescence microscope and the images were analyzed by ImageJ software.

3) Transmission electron microscopy:

1-3 mm of tissue was taken from the ischemic site of the left ventricle quickly after the mice were executed, and the tissue was pre-fixed with 2% glutaraldehyde for 2 hours at 4°C, rinsed with 0.1 M sodium dimethylarsenate, and post-fixed with 4% osmium acid for 2 hours. After graded ethanol dehydration, epoxy resin infiltration, embedding and polymerization hardening, preparation of 50–70 nm ultrathin sections, after double staining with uranyl acetate and lead citrate, ultrastructural changes were observed in transmission electron microscopy.

4) Determination of ROS level in LV myocardial tissue:

Frozen myocardial tissue sections were taken out from −80°C, naturally dried and washed with PBS, incubated with 5 uM DHE dye at 37°C for 30 minutes away from light, washed with PBS and sealed with DAPI-containing fluorescent sealer, and then observed and photographed under the fluorescence microscope to analyze the expression level of ROS in the tissues.

### Measurement of LV myocardial IS and AAR in mice

After 12 hours of reperfusion, each mouse was anesthetized again and the chest cavity was opened. The ascending aorta was cannulated and perfused with saline to flush the blood. The left anterior descending branch was occluded again with the same suture at the ligation site. To visualize the AAR, 1% Evans blue dye was injected into the aorta. Subsequently, the heart was excised and washed with PBS. The hearts were frozen at −20°C for 1 hour, sliced into 2.5-mm-thick cross-sections, and incubated at 37°C for 15 minutes after addition of 1% TTC solution, followed by termination of the reaction with 4% paraformaldehyde. IS (light color) and AAR (red color) were measured on both sides of each section using Image-Pro Plus 6.0 software and averaged, and the same method was used to calculate the values for each section, calculate the mean, and calculate the percentage of IS and AAR for the section (n = 8).

### Cardiac echocardiography in mice

Mice were anesthetized with 2% isoflurane before execution (12 hours of reperfusion), and cardiac structure and function were assessed by transthoracic echocardiography (VisualSonics VeVo 2100 Imaging System, Toronto, Canada). Two-dimensional targeted M-mode tracings were recorded from the mid papillary muscle in parasternal short-axis view and the lower papillary muscle in parasternal long-axis view, and the minimum of six consecutive cardiac cycles were acquired and analyzed to determine the left ventricular end-diastolic volume (LVEDV), left ventricular end-diastolic internal diameter (LVEDID), and left ventricular end-diastolic internal diameter (LVEDI). ventricular end-diastolic diameter (LVEDD), and Left Ventricular End-Systolic Volume (LVESV), Left ventricular end-systolic diameter, LVESD). Finally, EF and FS were calculated using the formulas, EF=(LVEDV-LVESV)/LVEDV×100% and FS=(LVEDD-LVESD)/LVEDD×100%.

### Serum biochemical indexes

Blood was taken from mice after execution, and serum was collected after centrifugation. Serum lactate dehydrogenase (LDH), malondialdehyde (MDA), glutathione (GSH) (BiyunTian, China) as well as myocardial injury-related markers creatine kinase (CK) (Nanjing Jianjian, China), creatine kinase isoforms (CK-MB), high-sensitivity troponin (Hs-Tn), and pro-brain natriuretic peptide (pro-BNP) levels (Jiangsu Jingmei, China) were tested according to the instructions of the manufacturer’s kit to assess the degree of myocardial injury and oxidative stress.

### RNA extraction and RT-qPCR assay

Myocardial tissues or cells treated with DEPC were lysed by adding Buffer R-I lysate into the grinding tube, and Buffer R-II and isopropanol were added to precipitate after automatic grinding and homogenization, and the supernatant was washed with Buffer W1 and W2 after transmembrane, and the purified RNA was finally obtained, and was stored at −80°C for spare use. The cDNA was synthesized according to the one-step gDNA removal kit from Beijing All-Style Gold Biotechnology Company. The concentration and purity of RNA were detected by UV spectrophotometer. After reverse transcription and synthesis of cDNA, real-time fluorescence quantitative PCR (RTFQPCR) was carried out to detect the expression of target genes by SYBR Green dye method, and melting curve analysis was carried out to verify the specificity of the amplification products after PCR was completed. The target genes and their primer sequences were shown in **S2 Table**.

### Protein extraction and Western blot assay

Total protein was collected by centrifugation after tissue or cell lysis, and protein concentration was determined by BCA method. Samples were separated by SDS-PAGE gel electrophoresis, transferred to PVDF membrane, washed by TBST and closed with 10% skimmed milk powder for 2 h. After addition of target protein primary antibodies (including cGAS, STING, IRF3, p-IRF3, NLRP3, Caspase-1, ASC, IL-1β, IL-18, GSDMD, Tubulin) (Abmart, China) was incubated overnight at 4°C, and the HRP-labeled secondary antibody was incubated for 1 hr after TBST washing on the following day. After washing again and incubation with ECL luminescent reagent, the target proteins were visualized in a chemiluminescent imaging system to detect changes in the expression levels of the target proteins.

### Cell culture and lentivirus-mediated overexpression and interference in the construction of stable screening strains

Lentivirus-mediated stable screening strain construction and overexpression stable screening strain construction were purchased from Suzhou GenePharma Company. Specifically, AC16 cells were cultured in DMEM + 10% FBS at 37 °C, 5% CO₂ with saturated humidity, and passaged at 70–80% confluence. For transduction, cells were seeded in 6-well plates and infected overnight with LV5-EF1α-GFP-Puro–STING (third-generation packaging; VSV-G pseudotyped) in complete medium containing polybrene 5 µg/mL. Medium was replaced the next day; after 48–72 h, cells were transferred to 60-mm dishes and selected with puromycin 1.0 µg/mL for ≥7 days to obtain stable pools. Transduction efficiency was monitored by GFP, and STING overexpression was verified by RT-qPCR (**see** **S5A Fig**). shRNAs targeting human STING were cloned into LV3-H1-GFP-Puro; virus was produced with a third-generation, four-plasmid system. AC16 cells were transduced as above (polybrene 5 µg/mL, overnight), recovered 48–72 h, then selected with puromycin 1.0 µg/mL for ≥5–7 days to generate stable pools. Knockdown was confirmed by RT-qPCR (**see** **S5B Fig**). The guide (target) sequences (5′ → 3′) for all shRNAs and the scramble control are listed in Supplementary **S3 Table**.

### H/R cell model

AC16 cells were cultured to a certain level of fusion, and the old DMEM complete medium was aspirated and discarded and replaced with 8 mL of sugar-free medium, after which the cells were cultured under hypoxic conditions. The hypoxic environment was achieved by using an anaerobic culture box (2.5 L) (Mitsubishi, Japan) equipped with an anaerobic gas-producing bag (Mitsubishi, Japan) and an oxygen indicator (Mitsubishi, Japan), which reduces the oxygen concentration to less than 0.1% within 1 hour. For the Control group, the cells were similarly switched to 8 mL of sugar-free medium, and the cells continued to be cultured in a conventional CO₂ incubator, maintaining the standard conditions of 37°C and 5% CO₂. After 6 h of hypoxia, the cells were removed from the hypoxic condition, the medium was replaced (completely), and the incubator was incubated for 3 h for subsequent experiments. The Control group was treated similarly.

### Statistical analysis

Statistical analysis was performed using SPSS 20.0 and R (v4.3.0). Normality and homogeneity of variances were assessed using the Shapiro–Wilk test and Levene’s test, respectively. Data are presented as mean ± SD. For comparisons among ≥3 independent groups, one-way ANOVA followed by Tukey’s multiple comparisons test was used when assumptions were met; Welch’s ANOVA was used when variances were unequal. When data were clearly non-normally distributed or when sample size was small, the Kruskal–Wallis test followed by Dunn’s post hoc test with Holm adjustment was applied. For two independent groups, an unpaired two-sided t test was used for approximately normal data; otherwise, the Wilcoxon rank-sum test was used. Proteomics intensities were log2-transformed prior to analysis and analyzed using two-sided unpaired t tests as specified. Correlations were assessed using Spearman’s rank correlation. For high-dimensional correlation screening and other gene-level analyses, false discovery rate (FDR) was controlled using the Benjamini–Hochberg procedure; both nominal P values and BH-adjusted q values are reported in **S4 Table**. All tests were two-sided and P < 0.05 was considered statistically significant unless otherwise stated. Sample collection, processing and analyses were performed by independent investigators, and the analyst was blinded to group allocation during data processing.

## Results

### Transcriptome analysis identifies pyroptosis-related gene signatures in I/R

**Fig 1:**
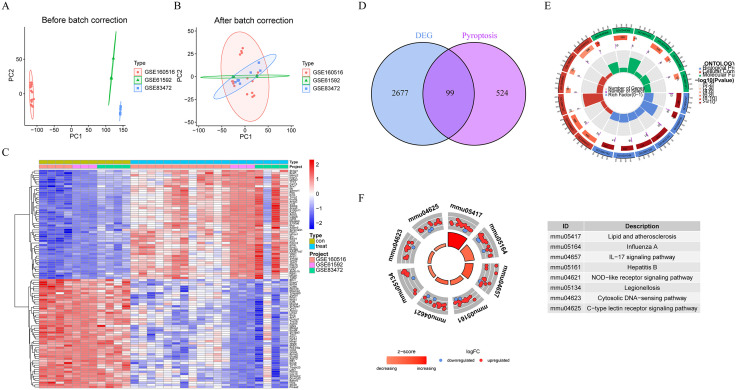
Identification and enrichment analysis of pyroptosis-related gene signatures in myocardial ischemia-reperfusion injury (I/R). **(A-B)** Principal Component Analysis (PCA) visualizations demonstrate effective removal of batch effects across multiple I/R datasets using the ComBat algorithm, with clear clustering observed before (A) and after (B) batch correction. **(C)** Heatmap representation of the top 50 significantly differentially expressed genes identified by limma analysis, highlighting clear distinctions between control and I/R groups (rows: genes; columns: samples; scaled expression shown as Z-scores) (included 11 control samples and 19 MI/R samples). **(D)** Venn diagram illustrating the intersection of 2776 I/R-associated differentially expressed genes and known pyroptosis-related genes, identifying 99 key pyroptosis-associated genes involved in I/R injury. **(E)** Gene Ontology (GO) functional enrichment analysis showing significant enrichment of differential genes in inflammation related biological processes. **(F)** Kyoto Encyclopedia of Genes and Genomes (KEGG) pathway analysis highlighting significant pathways. Abbreviations: DEG, differentially expressed gene; GO, Gene Ontology; KEGG, Kyoto Encyclopedia of Genes and Genomes.

The ComBat algorithm effectively removed batch effects across multiple I/R datasets (**Fig 1A**,**1B**). We screened for I/R-associated 2776 differential genes by limma, and the heatmap highlighted 50 genes that were significantly up- and down-regulated after I/R (**Fig 1C**). Further, we obtained a total of 99 I/R-associated cellular pyroptosis genes by taking intersections with cellular pyroptosis genes (**Fig 1D**). GO functional enrichment showed that the differential genes were significantly enriched for the regulation of inflammatory response, regulation of apoptosis signaling pathway, intrinsic apoptosis signaling pathway, I-kappaB kinase/NF-kappaB signaling, regulation of interleukin-1 production, etc. Inflammation-related pathways (**Fig 1E**). KEGG enrichment analysis also suggested that Lipid and atherosclerosis, IL-17 signaling pathway, NOD-like receptor signaling pathway, and Cytosolic DNA-sensing pathway may be associated with I/R-related pyroptosis signaling (**Fig 1F**).

### Single-cell gene expression profiles after I/R

**Fig 2:**
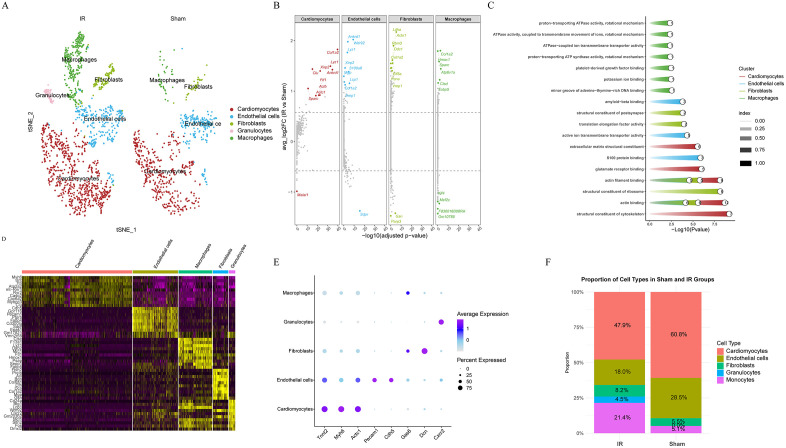
Single-cell transcriptome analysis reveals different cell populations and different gene expression after myocardial ischemia/reperfusion (I/R) injury. **(A)** The t-SNE embedding showing major cardiac cell types (cardiomyocytes, fibroblasts, endothelial cells, monocytes/macrophages, granulocytes). **(B)** Dot plot showing differentially expressed genes found in cardiomyocytes, fibroblasts, endothelial cells, and granulocytes in the Sham and I/R groups, highlighting key marker genes (dot size = cell-level detection rate; color = average expression). **(C)** GO enrichment analysis of differentially expressed genes in different cells. **(D)** Heatmap depicting expression levels of marker genes in different cell types, illustrating different transcriptional profiles. **(E)** Violin plot confirming the distribution and expression levels of known cell type-specific marker genes: **(F)** Bar graph showing changes in cell population proportions, with a significant decrease in cardiomyocytes and endothelial cells and a significant increase in monocytes/macrophages and granulocytes in the I/R group compared with the Sham group. Abbreviations: DEG, differentially expressed gene; GO, Gene Ontology; I/R, ischemia–reperfusion.

We first performed unsupervised graph clustering (SNN/Louvain) on the adjacency matrix obtained after PCA dimension reduction in Seurat, yielding several cell clusters; subsequently, we used SingleR for reference-driven cell type annotation; finally, we visualized the results using t-SNE. Based on this, cells were annotated into major groups such as cardiomyocytes, fibroblasts, endothelial cells, monocytes/macrophages, and granulocytes (**Fig 2A**). Notably, the Granulocytes cell population was only present in the I/R group, probably due to its rapid aggregation in acute inflammation for phagocytosis/release of inflammatory factors. The distribution of marker gene expression of the common types of cells in the early stages of I/R demonstrated the accuracy of the annotation (**Fig 2E**) (Cardiomyocytes: “Tnnt2”, “Myh6”, “Actc1”; Endothelial cells: “Pecam1”, “Cdh5”; Macrophages: “Gas6”; Fibroblasts: “Dcn”; Granulocytes: “Cxcr2”) Significant marker genes were seen in **Fig 2D**. significant reduction in cardiomyocytes, endothelial cells, and significant increase in the proportion of monocytes/macrophages and granulocytes were observed in the IR group as compared to the Sham group (**Fig 2F**). Differential comparative analysis screened for differentially expressed genes in cardiomyocytes, fibroblasts, endothelial cells, and granulocytes between the I/R and Sham groups, and enrichment analysis was performed separately (**Fig 2B**). Differential genes in cardiomyocytes were significantly enriched in structural constituent of cytoskeleton, actin binding, actin filament binding, glutamate receptor binding, and other functional entries (**Fig 2C**).

### Single-cell sequencing reveals pyroptosis activity and communication patterns in different cell populations of the heart

**Fig 3:**
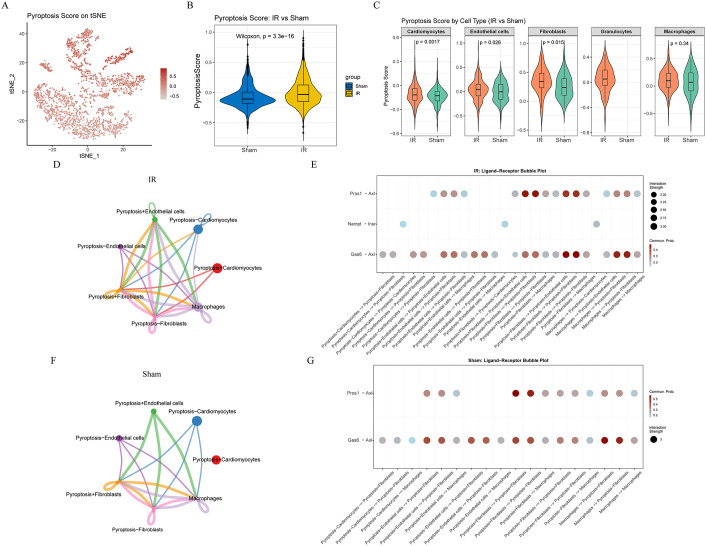
Single-cell analysis of pyroptosis (pyroptosis) activity and intercellular communication patterns in myocardial ischemia-reperfusion (I/R) injury. **(A)** t-SNE plot illustrating the distribution of pyroptosis-scores across single cells from heart tissues, highlighting variations in pyroptosis activity between Sham and I/R groups. **(B)** Boxplot quantitatively comparing overall pyroptosis-scores between the Sham and I/R groups, showing significantly increased pyroptosis activity in I/R group. **(C)** Pyroptosis-scores specifically analyzed across various cell types (cardiomyocytes, endothelial cells, fibroblasts, macrophages). **(D-F)** Circle plots depicting differential intercellular communication networks between pyroptosis-positive (pyroptosis^+^) and pyroptosis-negative (pyroptosis^−^) subpopulations of cardiomyocytes, endothelial cells, and fibroblasts. **(E-G)** Heatmap and chord diagrams illustrating specific receptor-ligand interactions mediating enhanced cellular communication in pyroptosis^+^ populations.

Based on the 99 genes obtained from the screening that constituted the set of pyroptosis-related genes, we calculated the cellular scotopic death score of each cell and plotted its distribution on the tSNE map (**Fig 3A**). Overall, the pyroptosis-score was significantly higher in the IR group than in the Sham group (P < 0.05; **Fig 3B**). Panel **Fig 3C** demonstrates the pyroptosis-score of different cell types, with cardiomyocytes, endothelial cells, and fibroblasts exhibiting higher pyroptosis-score than those of the Sham group, suggesting that these immune cells are in a highly inflammatory activated and pyroptosis susceptible state after ischemia-reperfusion. Macrophages, on the other hand, also tended to have higher pyroptosis-score, although not significantly so. By the median value of the pyroptosis-score, we classified cardiomyocytes, endothelial cells, and fibroblasts into pyroptosis^+^cardiomyocytes and pyroptosis^-^cardiomyocytes, pyroptosis^+^endothelial cells, pyroptosis^-^endothelial cells, pyroptosis^+^fibroblasts, and pyroptosis^-^fibroblasts, respectively. Cell communication was analyzed to disentangle different whether there was a difference in cellular communication in the state of pyroptosis activity. Compared with the Sham group, pyroptosis^+^fibroblasts in the I/R group enhanced the number of communications to pyroptosis^+^endothelial cells, and pyroptosis^+^cardiomyocytes also increased the number of communications to pyroptosis^+^fibroblasts (**Fig 3D-3F**). Receptor-ligand pair analysis revealed this increase in communication, which may be mediated by Pros1-Axl and Nampt-Lnsr (**Fig 3E-3G**).

### NLRP3 and Sting possess expression and functional correlations in I/R

**Fig 4:**
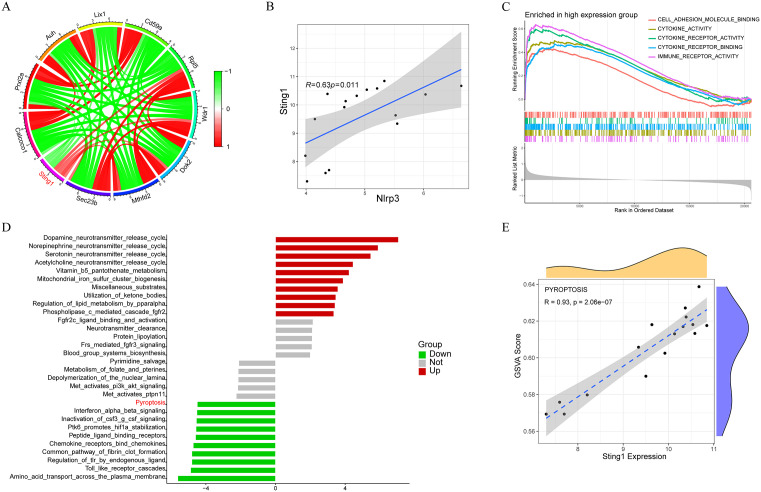
Co-expression and functional correlation analysis of NLRP3 and STING1 in myocardial ischemia-reperfusion (I/R) injury. **(A)** Volcano plot showing genes significantly correlated with Nlrp3 expression based on Spearman correlation analysis. **(B)** Correlation scatter plot specifically demonstrating the positive correlation between Nlrp3 and Sting1 gene expression. **(C)** Gene Set Enrichment Analysis (GSEA) comparing the Sting1 high-expression and Sting1 low-expression groups. **(D)** Gene Set Variation Analysis (GSVA) demonstrating pathway-based scoring differences, indicating significant enrichment of pyroptosis-associated signaling in the Sting1 low-expression group, **(E)** Strong positive correlation between Sting1 expression and pyroptosis-related signaling pathways. Abbreviations: GSEA, gene set enrichment analysis; GSVA, gene set variation analysis; FDR, false discovery rate.

Due to the key role of Nlrp3 in I/R, we explored the co-expression network of Nlrp3, based on Spearman correlation, we got a total of 1,375 relevant and highly correlated genes based on the screening criteria (|R| > 0.6, P < 0.05), of which, Wdrl, Dok2 and so on possessed a high degree of correlation, which included Sting1 (**Fig 4A-4B**). Based on the expression of Sting1, we divided the Sting1 high-expression group and Sting1 low-expression group and performed GSEA enrichment analysis, and found that cell adhesion molecule binding, cytokine activity, and cytokine receptor activity and binding were significantly up-regulated in the high-expression Sting1 group (**Fig 4D**). We further performed GSVA analysis, a pathway-based scoring algorithm, and found that an enrichment of pyroptosis-related signaling occurred in the Sting low expression group, and pyroptosis-related signaling pathway scoring showed a highly positive correlation with Sting1 expression (R = 0.93; P < 0.05; **Fig 4E**).

### I/R modeling and proteomics confirm high correlation between STING and NLRP3

**Fig 5:**
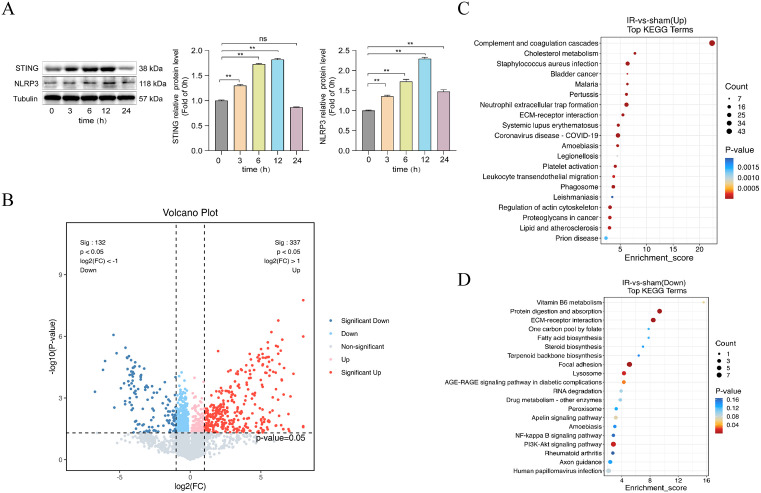
Western blot and proteomic analysis confirm dynamic co-regulation of STING and NLRP3 during myocardial ischemia-reperfusion (I/R). **(A)** Western blot showing the dynamic expression of STING and NLRP3 proteins in mouse heart tissues at different time points post-reperfusion (0 h, 3 h, 6 h, 12 h, and 24 **h)** (n = 3). **(B)** Volcano plot identifying 469 significantly altered proteins (337 upregulated, 132 downregulated) in the I/R group based on LC-MS/MS proteomic analysis (n = 3). **(C)** KEGG pathway enrichment analysis of upregulated proteins. **(D)** KEGG enrichment analysis of downregulated proteins.

Pre-experiments were performed using Western Blot to examine the changes in the expression of STING and NLRP3 proteins at different time points (0 h, 3 h, 6 h, 12 h and 24 h) after myocardial reperfusion in mice in order to determine the dynamic pattern of their changes. As shown in **Fig 5A**, the expression of STING proteins gradually increased with the prolongation of reperfusion time, reached a peak at 12 h, and then decreased at 24 h, indicating that the expression patterns of STING and NLRP3 were closely related to the process of myocardial reperfusion, which provided a basis for the determination of the optimal time point of reperfusion in the subsequent experiments. Subsequently, proteomic analysis of mouse myocardial I/R myocardial tissues was performed using LC-MS/MS technology, and a total of 5000 plausible proteins were identified. Differential proteins were screened by combining volcano and cluster plots, and a total of 469 significantly different proteins were identified according to the screening criteria FC ≥ 1.2 and P < 0.05. 337 up-regulated proteins and 132 down-regulated proteins were identified in the I/R group (**Fig 5B**). KEGG enrichment analysis showed that the up-regulated proteins correlated with Neutrophil extracellular trap formation, Leukocyte transendothelial migration and cholesterol metabolism. While down-regulated proteins were associated with Lysosome, RNA degradation, PI3K-Akt signaling, NFκB signaling (**Fig 5C-5D**).

### STING intervention affects cardiac function, oxidative stress and NLRP3 inflammasome activation in I/R mice

**Fig 6:**
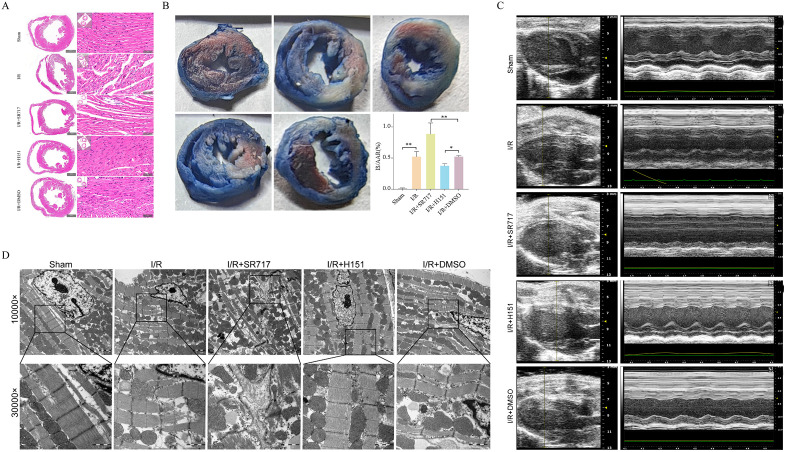
STING modulates myocardial structural injury and cardiac function in mice subjected to ischemia-reperfusion (I/R). **(A)** Representative H&E staining of left ventricular myocardium in each group (n = 8). **(B)** Quantification of infarct size (IS) relative to area at risk (AAR) based on TTC/Evans Blue staining (n = 8). **(C)** Left ventricular ejection fraction (EF%) assessed by echocardiography (n = 8). **(D)** Representative transmission electron microscopy images showing myocardial ultrastructure (n = 8).

We verified the effects of STING on myocardial ischemia-reperfusion injury by in vivo experiments in mice. After the successful establishment of the I/R model, H&E staining showed that myocardial fibers in the Sham group were neatly aligned, with clear transverse striations and intact myocyte nuclei, while the left ventricular myocardium in the I/R group showed obvious damage features, with a large number of myocytes undergoing degeneration and necrosis, nuclear condensation and fracture, and structural disorders of the myofibrils, and myocardial damage in the I/R + SR717 group was even greater than that in the I/R group Myocardial necrosis was exacerbated by STING hyperactivation (**Fig 6A**), as evidenced by the significant reduction of cardiomyocytes, the thickly stained nuclei of the remaining cells, consolidation, and the near disappearance of myofibrillar structures. Cross-sections of mouse myocardium stained with TTC+Evans blue were used to assess the extent of I/R injury.IS/AAR values were significantly higher in the I/R group than in the Sham group, and were significantly higher in the SR717-treated than in the I/R+DMSO group, whereas they were significantly lower in the H151-treated than in the I/R+DMSO group (P < 0.05; **Fig 6B**). Echocardiographic results showed that mice in the I/R group had significantly lower left ventricular ejection fraction (EF) (P < 0.05) and impaired cardiac function compared with the sham-operated group. The EF of the I/R + SR717 group given the STING agonist SR717 was further decreased, which was significantly different from that of the I/R+DMSO group (P < 0.05), suggesting that the activation of STING deteriorated cardiac function; on the contrary, the EF value of the I/R + H151 group applying the STING inhibitor H-151 was significantly improved compared with that of the I/R+DMSO group (P < 0.05), and the cardiac function was improved (**Fig 6C**). Under the ultrastructure, the cardiac muscle fibers in Sham group were neatly arranged, and the mitochondria were evenly distributed between the cardiac muscle fibers, which were regularly arranged and structurally intact. In the I/R and I/R+DMSO groups, myocardial fibers were not neatly aligned, partially broken, mitochondria were disorganized, and mitochondrial membrane and mitochondrial cristae were blurred. Myocardial fibers in the I/R + SR717 group also showed disorganized alignment, with part of the myocardial fibers broken and lysed, and mitochondria were disorganized with blurred structure of mitochondrial membrane and cristae. In contrast, in the I/R + H151 group, myocardial fibers were more neatly arranged, and mitochondrial membrane and cristae structures were clear (**Fig 6D**). Taken together, the histological and ultrastructural results further confirmed that activation of STING aggravated ischemia-reperfusion-induced myocardial morphologic injury, whereas inhibition of STING helped to maintain the structural integrity of cardiomyocytes.

Next, we examined several indicators of oxidative stress and myocardial injury. Compared with the Sham group, the level of ROS in myocardial tissues of mice in the I/R group was significantly higher (**S1A Fig**), and the levels of oxidative damage products such as LDH and MDA in serum were significantly higher than that of the control, whereas the level of the endogenous antioxidant GSH was significantly decreased (P < 0.05); at the same time, the levels of cardiac damage markers CK, CK-MB, hs-cTn, and pro-BNP were all were substantially higher than those in the control group (P < 0.05), indicating that reperfusion led to severe oxidative stress and myocardial cell injury. Compared with the I/R+DMSO group, the I/R + SR717 group showed more intense oxidative stress: enhanced ROS-positive signals, further elevation of LDH and MDA, and lower GSH content, and myocardial necrosis indices (CK systemic enzyme, cTn, and pro-BNP) were significantly higher than that of the I/R group without SR717 treatment (P < 0.05). On the contrary, in the I/R + H151 group, the above oxidative damage and myocardial necrosis indexes were alleviated: the ROS level was reduced, the serum LDH and MDA levels were decreased, whereas GSH was significantly regained; cardiac tethering enzymes and pro-BNP were also significantly lower than that in the I/R control group (P < 0.05) (**S1B-C Fig**). These results demonstrated that activation of STING exacerbates I/R-induced oxidative stress imbalance and myocardial injury, and inhibition of STING has some cardioprotective effects.

**Fig 7:**
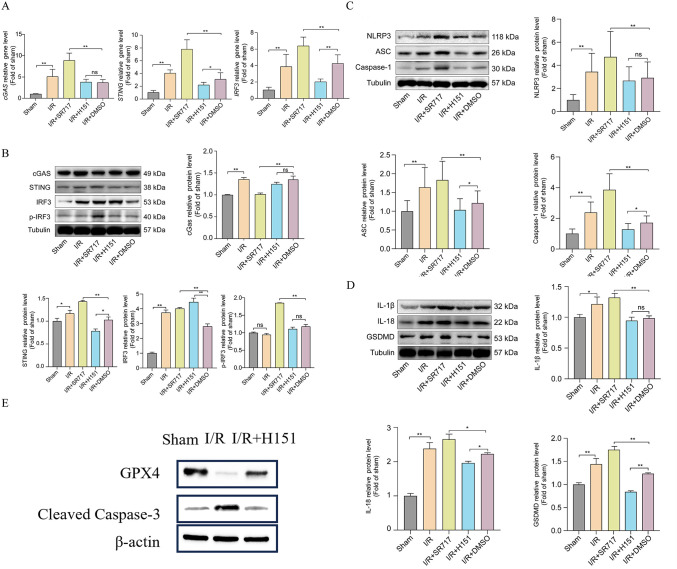
STING activation enhances the expression of pyroptosis-related genes and NLRP3 inflammasome components in I/R myocardial tissue. **(A)** RT-qPCR analysis of key genes in the cGAS-STING pathway (cGAS, STING, IRF3) and NLRP3 inflammasome (NLRP3, ASC, Caspase-1, IL-1β, IL-18, and GSDMD) in left ventricular myocardium (n = 8). **(B)** Western blot analysis of key genes in the cGAS-STING pathway (cGAS, STING, IRF3) in left ventricular myocardium (n = 3). **(C-D)** Western blot analysis of key genes in the NLRP3 inflammasome (NLRP3, ASC, Caspase-1, IL-1β, IL-18, and GSDMD) in left ventricular myocardium. Abbreviations: SR-717, non-nucleotide STING agonist (cGAMP-mimetic); H-151, covalent STING antagonist that blocks STING palmitoylation; DMSO, dimethyl sulfoxide (vehicle) (n = 3). **(E)** Western blot analysis revealed that STING expression is associated with increased Caspase-3and potentially regulates GPX4 (n = 3).

At the molecular level, we examined the changes in the expression of STING pathway and NLRP3 inflammasome-associated factors. qPCR and WB results showed that the expression of multiple inflammation- and pyroptosis-associated genes was significantly up-regulated in LV myocardial tissues of mice in the I/R group (**Fig 7A-7B**). I/R led to an increase in the expression of cGAS, STING, and IRF compared to that in the Sham group, suggesting that the innate immune DNA-sensing pathway was activated. suggesting that the innate immune DNA-sensing pathway was activated; meanwhile, the expression levels of NLRP3, ASC, and Caspase-1, as well as downstream effector molecules GSDMD, IL-1β, and IL-18 were significantly elevated (P < 0.01; **Fig 7C-7D**; **S2 Fig**), indicating that NLRP3 inflammasome and their mediated pyroptosis after I/R was strongly induced. Compared with the I/R+DMSO group, STING agonist treatment (I/R + SR717 group) further pushed up the expression of the above genes: whereas the application of STING inhibitor (I/R + H151 group) produced the opposite effect. Recently, Wang X et al [31]. published findings on the potential link between Sting and apoptosis and ferroptosis. In addition to inducing NLRP3 inflammasome-associated pyroptosis markers, we observed that STING regulation was also associated with changes in markers related to other regulatory cell death programs. Specifically, STING activation was accompanied by increased cleaved Caspase-3 (an apoptosis-associated marker) and decreased GPX4 (an iron death-associated antioxidant defense), while STING inhibition showed the opposite trend (**Fig 7E**). Collectively, these data suggest that STING may participate in myocardial ischemia/reperfusion injury through multiple regulatory cell death pathways, with the STING-NLRP3 axis representing a key component of inflammatory pyroptosis.

### STING overexpression exacerbates H/R cardiomyocyte injury with effects dependent on NLRP3 inflammasome

**Fig 8:**
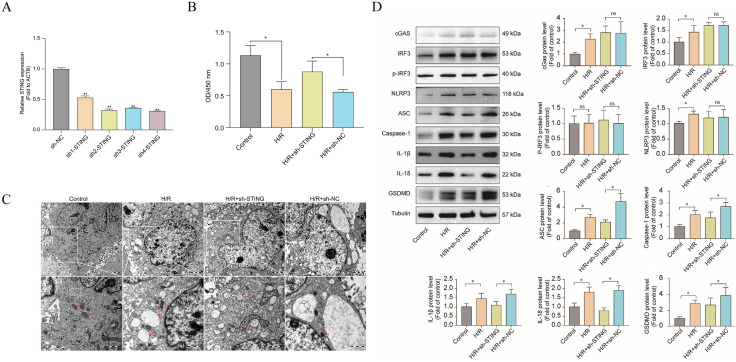
STING regulates H/R-induced cardiomyocyte injury. **(A)** Western blot analysis confirming the efficiency of STING knockdown in AC16 cardiomyocytes using shRNA constructs. sh4 was selected for subsequent experiments (n = 3). **(B)** Cell viability of AC16 cardiomyocytes under normoxia or hypoxia/reoxygenation (H/R) conditions (n = 3). **(C)** Transmission electron microscopy images showing cellular ultrastructure (n = 3). **(D)** Western blot quantification of NLRP3, Caspase-1, and IL-1β expression under different treatment conditions (n = 3).

Next, we further validated the causal relationship between STING and NLRP3 inflammasome at the cellular level. H/R modeling resulted in significant injury to AC16 cardiomyocytes, with a decrease in cell viability of approximately 30% in the H/R group compared with normoxic controls (P < 0.01; **Fig 8B**), ultrastructural damage (**Fig 8C**), and an increase in culture supernatant LDH and CK release increased (P < 0.05; **S3A-B Fig**), suggesting increased cell membrane permeability and cell necrosis. intracellular ROS generation in H/R cells was significantly higher than the control level (P < 0.01; **S3C Fig**). We constructed sh STING stable transient cell line and selected sh4 to be used as a subsequent cell model (**Fig 8A**), and overexpression of STING cell line (OE-STING), which were used to compare the effects of STING knockdown and overexpression on H/R cells. STING knockdown significantly increased cell viability, and cell membrane and organelle structures were observed to be more intact and mitochondrial structure was more complete in TEM compared with that of the H/R group, and the mitochondria were significantly higher than the control group. R group, and damage features such as mitochondrial swelling and endoplasmic reticulum expansion were alleviated as well as ROS levels were down-regulated (**Fig 8B-8C;** **S3B Fig**). The mRNA and protein expression of inflammasome-associated molecules such as NLRP3, Caspase-1, and IL-1β were significantly downregulated after STING knockdown (P < 0.05; **Fig 8D**; **S3E Fig**). Immunofluorescence quantification showed that the signal intensity of IL-1β and IL-18 was significantly lower than that of the H/R + sh-NC group (P < 0.05; S3C-D Fig) These in vitro experimental results were consistent with the in vivo trend.

**Fig 9:**
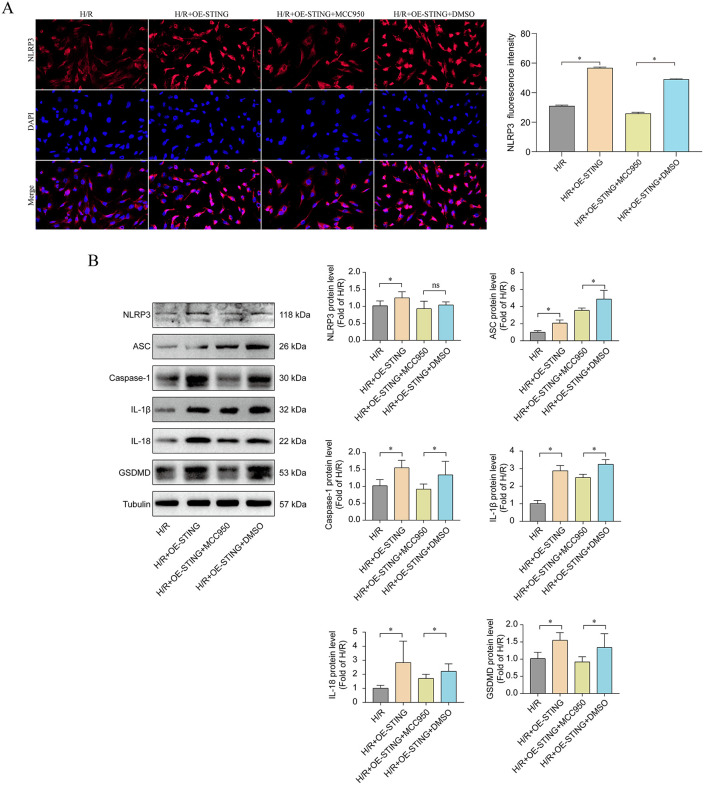
NLRP3 inhibition attenuates STING-induced pyroptosis and inflammation in H/R-treated cardiomyocytes. **(A)** Immunofluorescence images showing intracellular NLRP3 localization and fluorescence signalin H/R-treated AC16 cells overexpressing STING, with or without NLRP3 inhibitor MCC950 (n = 3). **(B)** Western blot analysis of key components of the NLRP3 inflammasome (NLRP3, ASC, Caspase-1) and downstream effector proteins (IL-1β, IL-18, GSDMD) in H/R + OE-STING cells. MCC950 significantly suppresses expression of these pyroptosis-related markers, confirming that STING-induced inflammatory damage is mediated via the NLRP3 inflammasome pathway (n = 3).

To further investigate the upstream-downstream linkage between STING and NLRP3, we explored the effect of the NRP3 inhibitor MCC950 on STING overexpressing cells. Upon addition of MCC950 to H/R + OE-STING cells, significant changes in inflammation and pyroptosis indicators could be observed: immunofluorescence results showed that the fluorescence intensity of intracellular NLRP3 spots in the MCC950-treated group was drastically reduced compared with that of the non-inhibitor-treated H/R + OE-STING+DMSO group (**Fig 9A**), and the expression of IL-1β and IL −18 expression was also significantly reduced (**S4A-B Fig**). MCC950 was able to effectively block the downstream effects induced by STING overexpression, significantly reducing the levels of key components of NLRP3 inflammasome (NLRP3, ASC, Caspase-1) as well as the end effector molecules (mature IL-1β, IL-18, and GSDMD) (P < 0.05; **Fig 9B**; **S4C Fig**). Together, these data support that STING exacerbates cardiomyocyte injury, at least in part, through NLRP3 inflammasome–associated pyroptosis. Blocking NLRP3 activation could partially reverse the detrimental effects of STING overactivation, thereby reducing the release of inflammatory factors and improving cell survival.

## Discussion

In this study, we provide multi-omics and experimental evidence that STING signaling is activated during MIRI and may contribute to injury, in part by promoting oxidative stress and NLRP3 inflammasome–associated pyroptosis. By integrating bioinformatic analyses with in vivo and in vitro cell-based experiments, we show that STING activation exacerbates inflammation and myocardial injury, whereas STING inhibition is cardioprotective.

First, we characterized the expression profiles of genes related to pyroptosis in I/R by transcriptome data mining. We observed significant upregulation of multiple genes related to the NLRP3 inflammasome and pyroptosis in myocardial I/R. These findings suggest activation of pyroptosis-related signaling during early ischemia–reperfusion stress. This is consistent with prior evidence showing increased NLRP3 inflammasome components and GSDMD expression in I/R models [9]. We further constructed a pyroptosis signature score, which supported a coordinated pro-pyroptotic molecular signature associated with I/R. Multigene signature scoring has been increasingly used in cardiovascular research to summarize pathway-level activity. Future studies are warranted to evaluate whether pyroptosis-related gene sets can assist in assessing inflammatory injury status in I/R. Of course, due to the small dataset, subsequent consideration could be given to pooling data from more cohorts to construct and validate a signature model for the 99 pyroptosis genes. Single-cell sequencing analysis provides a more intuitive perspective for understanding cellular heterogeneity and interactions in I/R. We found that in ischemia-reperfused cardiac tissues, cardiomyocytes, endothelial cells, and fibroblasts, among others, provided high scores of pyroptosis in I/R, suggesting that these cells may be the primary performers of pyroptosis. In cardiomyocytes, reperfusion can activate caspase-1 and cleave GSDMD via the NLRP3 inflammasome. This process promotes membrane pore formation and IL-1β/IL-18 release, thereby exacerbating contractile dysfunction and infarct expansion [32]. Endothelial cells also exhibit NLRP3-dependent pyroptosis under I/R conditions, and GSDMD-mediated death can increase vascular permeability and leukocyte adhesion, promoting microvascular obstruction and tissue ischemia [33,34]. Fibroblasts may undergo pyroptosis in response to danger signals, releasing pro-inflammatory cytokines that amplify local inflammation. They may also promote fibrosis through extracellular matrix remodeling, increasing myocardial stiffness and further impairing systolic–diastolic function [35]. Our cell–cell communication analysis further suggested that inflammatory mediators may signal through receptors on cardiomyocytes and fibroblasts, potentially contributing to secondary injury and additional cell death. Our inferred communication network suggests bidirectional signaling between immune cells and cardiomyocytes through the Pros1–AXL and NAMPT–Lnsr axes, which may facilitate propagation of inflammation and cell death. Pros1 released by immune cells may bind the AXL receptor on cardiomyocytes and activate downstream NF-κB signaling. This signaling can promote NLRP3 inflammasome priming and caspase-1 activation, thereby facilitating GSDMD-mediated pore formation and IL-1β/IL-18 release [36]. It has been shown that specific overexpression of Nampt in the myocardium exerts a protective effect in the I/R model by maintaining NAD⁺ levels, but in a high-inflammatory environment, exogenous Nampt instead promotes proinflammatory factor release and contributes to cell death [37]. This bi-directional Pros1-Axl-Nampt-Lnsr dialogue forms a “feedback amplifier” of the inflammatory cascade, which continuously activates the pyroptosis pathway at the cellular level, leading to impaired myocardial function and increased tissue remodeling.

In vivo experiments verified a critical role of STING in modulating inflammatory injury during MIRI. Administration of the STING agonist SR717 further deteriorated cardiac function in I/R mice compared with untreated I/R controls. In contrast, the STING inhibitor H-151 markedly improved cardiac function and reduced myocardial injury indices. This is consistent with the study of Hu et al [38], who reported that H-151 improved left ventricular function and attenuated myocardial fibrosis in a mouse infarction model. Our results further extend this finding by confirming the beneficial effects of STING inhibition in the dynamic process of ischemia-reperfusion. Mechanistically, we detected that the STING pathway (cGAS-STING-TBK1-IRF3) was significantly activated during I/R and induced elevation of a series of downstream inflammatory effectors, including type I interferons and inflammatory factors. Notably, STING activation showed a synchronous and enhanced trend with the activation of NLRP3 inflammasome. In SR717-treated mice, myocardial levels of NLRP3, caspase-1, and IL-1β were higher than in I/R controls, whereas H-151 suppressed the upregulation of these factors. This suggests that the STING pathway may promote the assembly and maturation of NLRP3 inflammasome through direct or indirect pathways. For example, STING-driven TBK1–IRF3 signaling may enhance inflammatory transcriptional programs that support NLRP3 inflammasome priming [39]. On the other hand, STING activation is often accompanied by mitochondrial damage and increased ROS production [40]. ROS is one of the important activation signals for NLRP3 inflammasome, and mitochondria-derived ROS can act as DAMP to drive inflammasome assembly [40]. Therefore, STING may also indirectly trigger the activation of NLRP3 inflammasome by exacerbating the oxidative stress environment. This inference was supported in our experiments: myocardial ROS content was highest in the SR717 group of mice, whereas ROS was inhibited in the H-151 group, which was positively correlated with the changes in NLRP3 activity in all groups. Previously, it was demonstrated that inhibition of the cGAS-STING pathway reduces the phosphorylation levels of TBK1 and IRF3 in diabetic myocardium, thereby attenuating the NLRP3 inflammasome-mediated inflammatory response [41]. We observed a similar pattern in the I/R model, supporting potential crosstalk and a feed-forward relationship between STING signaling and NLRP3 inflammasome activation.

In the AC16 cardiomyocyte H/R model, we directly regulated STING expression by genetic manipulation and confirmed the causal role of STING in promoting cardiomyocyte pyroptosis. STING overexpression significantly exacerbated H/R-induced cellular damage, including ultrastructural disruption, increased membrane permeability, and decreased viability. Meanwhile, NLRP3 inflammasome-associated metrics were dramatically increased in STING overexpressing cells, suggesting that excess STING signaling was sufficient to trigger endogenous inflammasome overactivation in cardiomyocytes. When STING was inhibited, the pyroptotic phenotype (including reduced NLRP3 speckle formation, decreased IL-1β/IL-18 release, and attenuated GSDMD-mediated membrane pore formation) was significantly alleviated, supporting STING as an upstream regulator of NLRP3 inflammasome–associated pyroptotic signaling in our models. In addition, we performed a “rescue” experiment using the NLRP3-specific inhibitor MCC950. The results were as expected: MCC950 treatment almost eliminated the inflammatory and death phenotype caused by STING overexpression, and inflammatory factor release and cellular ROS and edema were restored to near basal levels. This suggests that the effect of STING to exacerbate cardiomyocyte injury requires the mediation of NLRP3 inflammasome. This finding matches previous reports in other models, such as in the lipotoxic myocardial injury or tumor cardiotoxicity models [39,42], where both have found that inhibition of STING attenuates cardiomyocyte pyroptosis and thus exerts a protective effect. In the I/R context, our gain- and loss-of-function experiments provide direct evidence linking the STING–NLRP3 axis to cardiomyocyte pyroptosis, supporting this pathway as a candidate intervention target.

Our data and previous literature indicate that the cGAS-STING signaling pathway interacts with multiple regulatory cell death (RCD) mechanisms during myocardial ischemia-reperfusion. Previous studies have indicated that STING promotes apoptosis/necrosis in a context-dependent manner. Recent findings reveal that STING targets GPX4 for autophagic degradation, thereby exacerbating myocardial ischemia-reperfusion injury and amplifying ferroptosis in cardiomyocytes [31]. Consequently, during ischemia-reperfusion, pyroptosis and ferroptosis may coexist across different cardiomyocyte subtypes and temporal windows rather than being mutually exclusive. Further investigation into the crosstalk and synergistic mechanisms between these pathways will be of significant importance.

In summary, our study supports that STING activation contributes to I/R injury, at least partly through NLRP3 inflammasome activation, based on multi-dimensional evidence. On the one hand, targeting the STING pathway (e.g., STING inhibitor H-151 or upstream strategies to block cGAS) may represent potential approaches to reduce reperfusion injury; on the other hand, direct targeting of NLRP3 inflammasome (e.g., by applying MCC950) has been shown to significantly reduce pyroptosis and improve cardiac function. However, it is important to note that the present study mainly focused on the acute inflammatory injury stage in the early phase of ischemia-reperfusion and has not yet addressed the long-term prognosis and myocardial remodeling process. In addition, the conclusions we obtained at the cellular and mouse levels will need to be validated in the future in models that are closer to the clinic (e.g., large animal models or patient myocardial tissue samples).

## Supporting information

S1 Fig STING regulates oxidative stress and myocardial injury in I/R mice. (A) Representative images of DHE-stained heart sections showing ROS levels (*P < 0.05). (B) Quantitative expression of ldh, mda, and gsh among different groups. (C) Quantitative expression of serum biomarkers of myocardial injury: CK, CK-MB, hs-cTn and pro-BNP.
(TIF)

S2 Fig RT-qPCR analysis of key genes in the NLRP3 inflammasome (NLRP3, ASC, Caspase-1, IL-1β, IL-18, and GSDMD) in left ventricular myocardium. (TIF)

S3 Fig STING modulates oxidative stress, membrane damage, and inflammatory factor release in H/R-treated cardiomyocytes. (A) LDH and CK levels in the culture supernatant of AC16 cells. (B) Intracellular ROS levels measured by DHE staining. H/R induces ROS accumulation, which is further alleviated by STING knockdown. (C-D) Immunofluorescence images and quantification of IL-1β and IL-18 in AC16 cardiomyocytes. (E) RT-qPCR quantification of NLRP3, Caspase-1, and IL-1β expression under different treatment conditions.
(TIF)

S4 Fig MCC950 suppresses STING-induced inflammation and pyroptosis markers in H/R cardiomyocytes. (A-B) Immunofluorescence staining and quantification of IL-1β and IL-18 in H/R + OE-STING cells with or without MCC950. (C) RT-qPCR showing the expression of NLRP3, ASC, Caspase-1, IL-1β, IL-18, and cleaved GSDMD in different treatment groups.
(TIF)

S5 Fig Construction of lentivirus-mediated interference stable screening lines and qPCR validation of overexpression stable screening lines. (A) NC: Negative control group; A1716: Lentivirus ID for STING overexpression. (B) NC: Negative control group; K9362, K9520, K10834, K10835: Lentivirus IDs for different STING interference fragments.
(TIF)

S1 Table Pyroptosis-related genes from Genecards. (CSV)

S2 Table RT-qPCR target gene and its primer sequence. (XLSX)

S3 Table qPCR validation and shRNA target sequences. (XLSX)

S4 Table Correlation analysis of Nlrp3 with other genes. (XLSX)
