# Supplementary figures and images for: Multi-omics analysis reveals STING activation mediates NLRP3-related pyroptosis and exacerbates myocardial ischemia-reperfusion injury
Source: PLoS One. 2026 Feb 6;21(2):e0341839. doi: 10.1371/journal.pone.0341839 (PMC12880705; doi:10.1371/journal.pone.0341839)

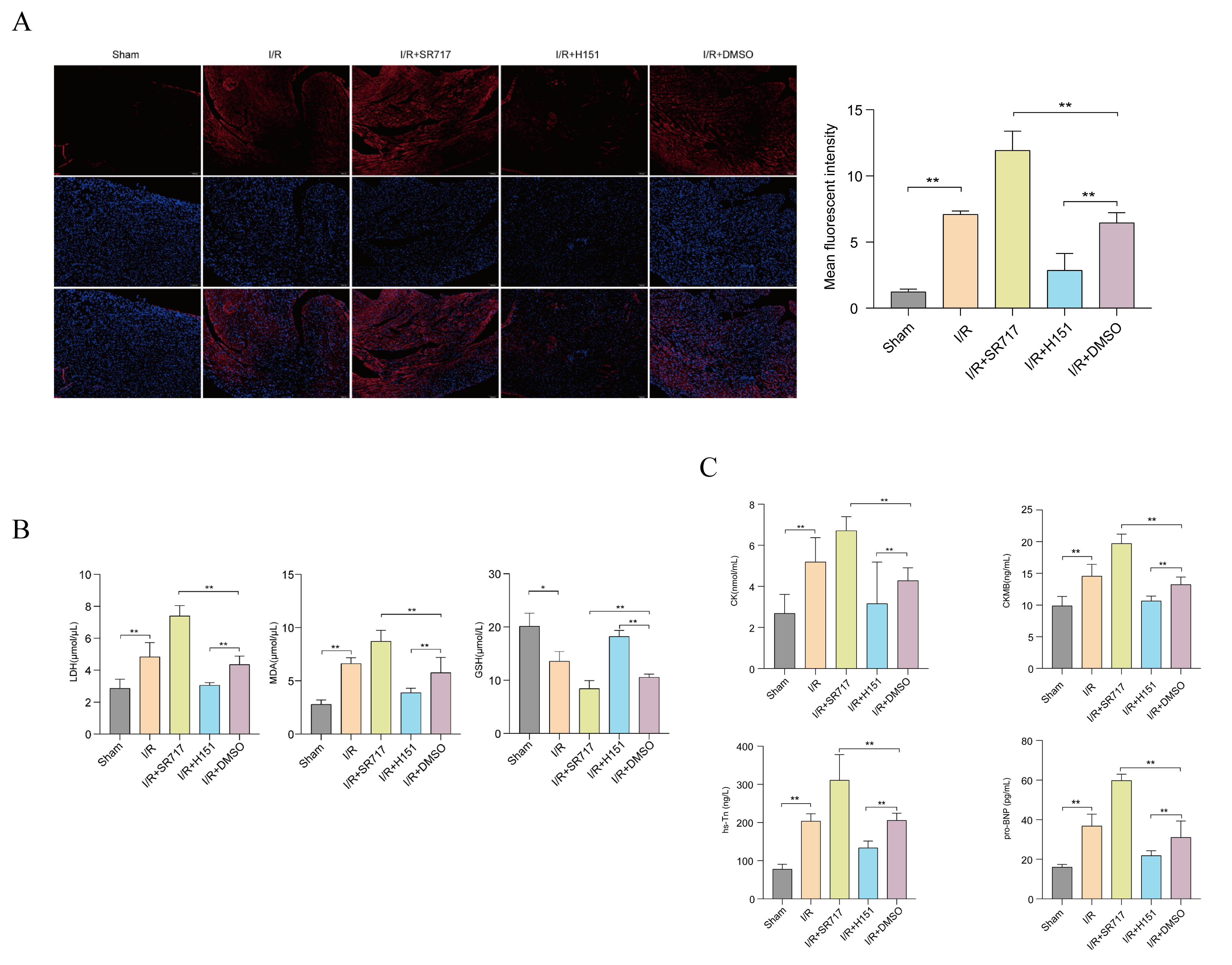

Supplement: S1 Fig — (A) Representative images of DHE-stained heart sections showing ROS levels (*P < 0.05). (B) Quantitative expression of ldh, mda, and gsh among different groups. (C) Quantitative expression of serum biomarkers of myocardial injury: CK, CK-MB, hs-cTn and pro-BNP. (TIF) [file pone.0341839.s001.tif]

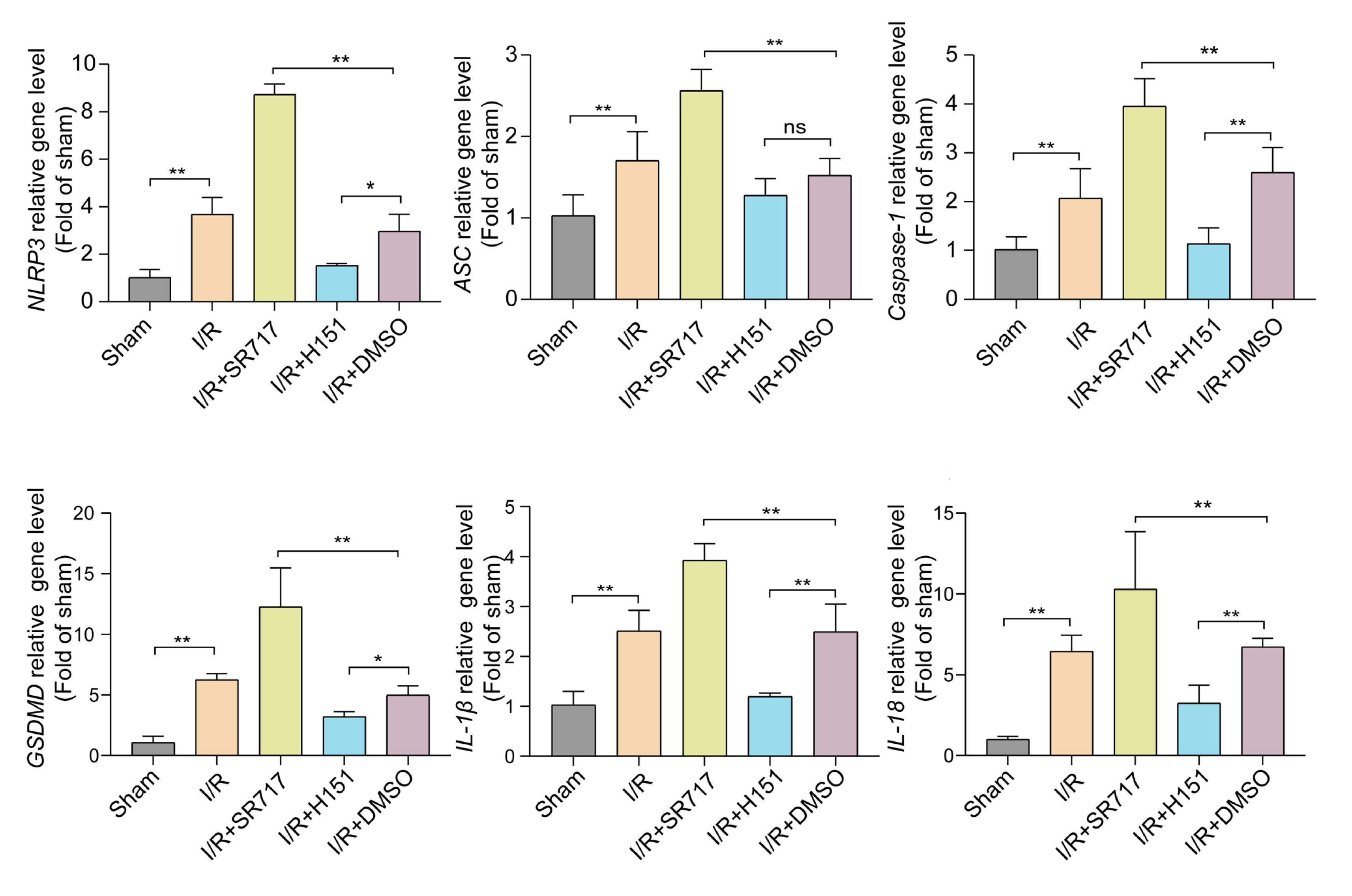

Supplement: S2 Fig — (TIF) [file pone.0341839.s002.tif]

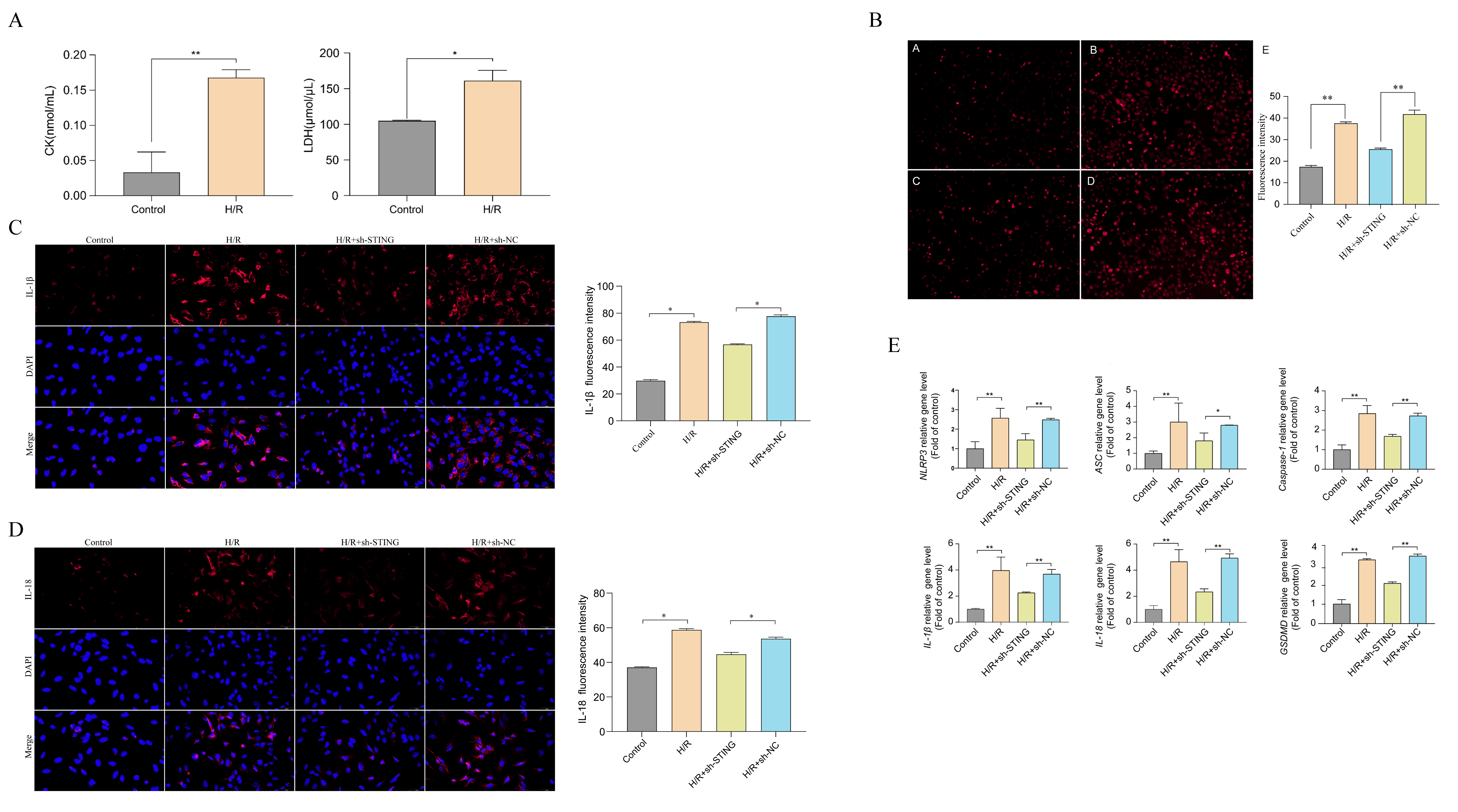

Supplement: S3 Fig — (A) LDH and CK levels in the culture supernatant of AC16 cells. (B) Intracellular ROS levels measured by DHE staining. H/R induces ROS accumulation, which is further alleviated by STING knockdown. (C-D) Immunofluorescence images and quantification of IL-1β and IL-18 in AC16 cardiomyocytes. (E) RT-qPCR quantification of NLRP3, Caspase-1, and IL-1β expression under different treatment conditions. (TIF) [file pone.0341839.s003.tif]

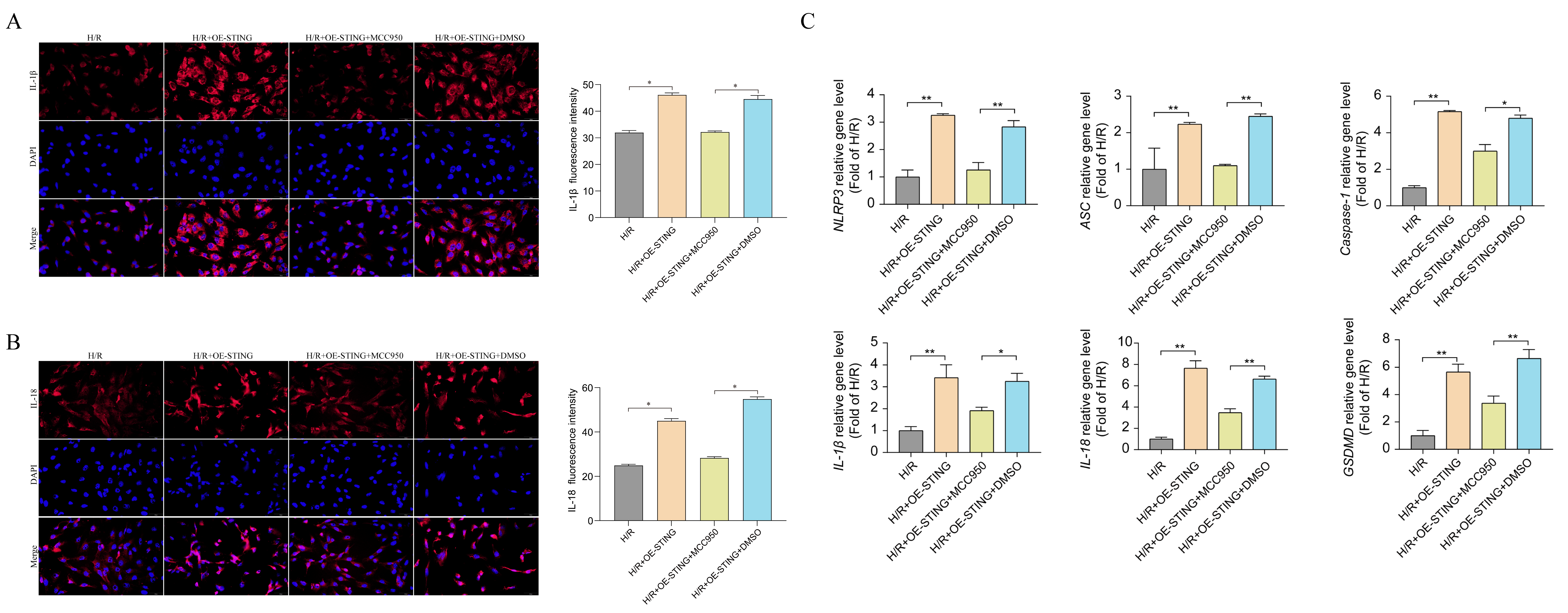

Supplement: S4 Fig — (A-B) Immunofluorescence staining and quantification of IL-1β and IL-18 in H/R + OE-STING cells with or without MCC950. (C) RT-qPCR showing the expression of NLRP3, ASC, Caspase-1, IL-1β, IL-18, and cleaved GSDMD in different treatment groups. (TIF) [file pone.0341839.s004.tif]

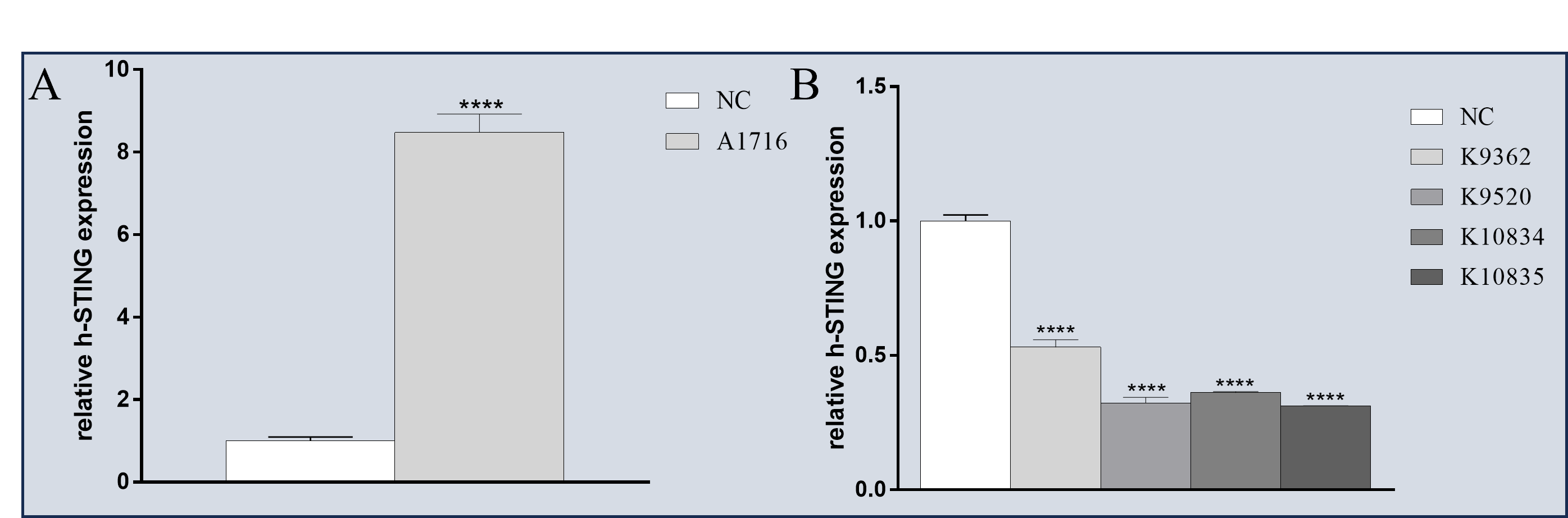

Supplement: S5 Fig — (A) NC: Negative control group; A1716: Lentivirus ID for STING overexpression. (B) NC: Negative control group; K9362, K9520, K10834, K10835: Lentivirus IDs for different STING interference fragments. (TIF) [file pone.0341839.s005.tif]
